# Cinnamon Extract Improves Insulin Sensitivity in the Brain and Lowers Liver Fat in Mouse Models of Obesity

**DOI:** 10.1371/journal.pone.0092358

**Published:** 2014-03-18

**Authors:** Tina Sartorius, Andreas Peter, Nadja Schulz, Andrea Drescher, Ina Bergheim, Jürgen Machann, Fritz Schick, Dorothea Siegel-Axel, Annette Schürmann, Cora Weigert, Hans-Ulrich Häring, Anita M. Hennige

**Affiliations:** 1 Department of Internal Medicine, Division of Endocrinology, Diabetology, Vascular Disease, Nephrology and Clinical Chemistry, Member of the German Center for Diabetes Research (DZD), University of Tuebingen, Germany; 2 German Center for Diabetes Research (DZD), Tuebingen, Germany; 3 Institute for Diabetes Research and Metabolic Diseases of the Helmholtz Center Munich at the University of Tuebingen (IDM), Tuebingen, Germany; 4 Department of Experimental Diabetology, German Institute of Human Nutrition, Potsdam-Rehbruecke, Germany; 5 Department of Nutritional Sciences, SD Model Systems of Molecular Nutrition, Friedrich-Schiller-University Jena, Jena, Germany; 6 Section on Experimental Radiology, Department of Diagnostic and Interventional Radiology, University of Tuebingen, Germany; University of Bremen, Germany

## Abstract

**Objectives:**

Treatment of diabetic subjects with cinnamon demonstrated an improvement in blood glucose concentrations and insulin sensitivity but the underlying mechanisms remained unclear. This work intends to elucidate the impact of cinnamon effects on the brain by using isolated astrocytes, and an obese and diabetic mouse model.

**Methods:**

Cinnamon components (eugenol, cinnamaldehyde) were added to astrocytes and liver cells to measure insulin signaling and glycogen synthesis. Ob/ob mice were supplemented with extract from *cinnamomum zeylanicum* for 6 weeks and cortical brain activity, locomotion and energy expenditure were evaluated. Insulin action was determined in brain and liver tissues.

**Results:**

Treatment of primary astrocytes with eugenol promoted glycogen synthesis, whereas the effect of cinnamaldehyde was attenuated. In terms of brain function *in vivo*, cinnamon extract improved insulin sensitivity and brain activity in ob/ob mice, and the insulin-stimulated locomotor activity was improved. In addition, fasting blood glucose levels and glucose tolerance were greatly improved in ob/ob mice due to cinnamon extracts, while insulin secretion was unaltered. This corresponded with lower triglyceride and increased liver glycogen content and improved insulin action in liver tissues. *In vitro*, Fao cells exposed to cinnamon exhibited no change in insulin action.

**Conclusions:**

Together, cinnamon extract improved insulin action in the brain as well as brain activity and locomotion. This specific effect may represent an important central feature of cinnamon in improving insulin action in the brain, and mediates metabolic alterations in the periphery to decrease liver fat and improve glucose homeostasis.

## Introduction

The pathogenesis of type 2 diabetes integrates obesity, insulin resistance, and finally insulin secretion failure. Besides the well-established approaches to improve insulin action and secretion in patients with type 2 diabetes, there is still a demand for alternative therapies. Traditional chinese medicinal plants have been screened and used as pharmacological active compounds for a long time to treat and prevent various chronic diseases, such as diabetes, atherosclerosis, cancer, aging, and other degenerative diseases [1]–[4]. In this regard it is worth to remind that one of the most popular diabetes medications, metformin, originates from the perennial herb *Galega officinalis* used in folk medicine to treat diabetes for hundreds of years [5].

One potential approach that arouses attention is cinnamon. Cinnamon extracts were shown to have antidiabetic effects as a number of cell studies demonstrated an insulin-like action. Additionally, cinnamaldehyde promoted glucose uptake into skeletal muscle through glucose transporter 4 translocation [6]. The treatment of diabetic subjects with cinnamon was investigated in several clinical trials. While a number of studies demonstrated an improvement in fasting blood glucose concentrations and insulin sensitivity [7]–[8], others did not show any beneficial effects [9]. Notably, cinnamon was not able to exert significant effects in type 1 diabetic patients while its insulin-like effects were present in type 2 diabetic patients [4]. Interestingly, cinnamon extract was described as beneficial in Alzheimer's disease by reducing β-amyloid oligomerization and cognitive decline [10]–[11], and cinnamon further prevented glutamate-induced neuronal death in cultured cerebellar granule cells [12].

The brain is the key organ to sense metabolic alterations and in turn controls food intake and glucose homeostasis, and there is considerable evidence that insulin is a key signal to act in the brain [13]. Insulin in the brain was shown to inhibit glucose production in the liver and therefore lowered blood glucose levels, while this was absent in insulin resistant states like high fat diet (HFD)- fed mice [14]–[15]. Cinnamon was never shown to affect insulin secretion *in vivo* [16], and we therefore reasoned that alterations in insulin action in the brain might contribute to the beneficial effect of cinnamon on glucose homeostasis through the brain-liver axis.

In previous studies we determined insulin action in the brain of lean and obese mice and correlated it to brain activity and locomotion. Insulin was able to increase cortical activity in lean animals while HFD-fed obese mice displayed insulin resistance [17]–[18]. As a readout of impaired insulin action in the brain, we established measures of insulin-mediated locomotor activity in mice and humans [17]. While normal-weight mice increased locomotor activity in response to intracerebroventricular (i.c.v.) insulin and therefore kept the balance between food intake and energy expenditure, insulin resistance as present in obese mice compromised brain and locomotor activity to lower blood glucose levels.

We and others showed that saturated free fatty acids cause insulin resistance in the human brain [19], and our previous mouse data speak to an effect of saturated fatty acids to impair brain activity [20], locomotion and to alter sleep architecture [18], while monounsaturated fatty acids even displayed beneficial effects in mice and humans. In another study, elevated leptin levels as present in obese subjects were shown to directly inhibit insulin action in the brain which finally led to physical inactivity in mice and humans [21]. So far, substances to increase or restore insulin action in the brain and improve brain activity, locomotion and favor glucose homeostasis are unknown. Stimulated by the observations suggesting brain effects of cinnamon, we hypothesized that the described effects of cinnamon extracts on fasting blood glucose might be dependent on a modulation of insulin action in the brain. Interestingly, earlier studies supposed that rather specific compounds of cinnamon extract than cinnamon itself exert beneficial effects. Therefore, we decided to use extract from *cinnamomum (C.) zeylanicum* that is regarded to be more effective and safe compared to cinnamon related species like *C. cassia* [22], where concentrations of coumarins are extremely high and cause health risks if consumed regularly in higher quantities [23].

First, we studied the *in vitro* effect of two major components of the essential oil obtained from the bark of *cinnamomum (C.) zeylanicum*: cinnamaldehyde and eugenol [24]–[25]. Cinnamaldehyde accounts for approximately 50–63% of the total composition [26]–[28]. For screening purposes in brain tissues, we first performed experiments in isolated murine astrocytes to substantiate our hypothesis on a brain effect of cinnamon. Astrocytes are the most abundant cell type of the human brain, and they are part of the blood-brain barrier [29], and exhibit the key enzymes that regulate synthesis and degradation of brain glycogen [30]. As important glial-neuronal interaction, astrocyte glycogen supplies neighboring neurons or axons with fuel under hypoglycemic conditions when delivery of blood glucose is insufficient to meet immediate energy requirements [31]–[32]. On the cellular level, we recently showed that astrocytes are insulin responsive and form glycogen upon insulin stimulation [33]. As we noticed altered responses on glycogen formation and insulin signaling by cinnamon compounds in primary murine astrocytes, we further evaluated cinnamon extract in mouse models *in vivo*. Together, the observed phenotype demonstrated beneficial effects of cinnamon in the brain that translate to less liver fat content and lower blood glucose concentrations in obese and diabetic mice.

## Methods

### Primary astrocyte cultures and glycogen synthesis

Two-day-old C57BL/6 mice pups were used for isolation of primary astrocytes as previously described [34]. Briefly, astrocytes were cultured in Dulbecco's Modified Eagle's Medium (DMEM) with 4.5 g/l glucose (Lonza, Brussels, Belgium). Prior to treatment, the medium was changed to glucose-deprived neuronal cell culture medium (1 g/l glucose) for 24 hours. Cells were pretreated with 50 μM cinnamaldehyde, 500 μM eugenol (both Sigma Aldrich, Germany) or with both substances for 2 h before stimulating them with 100 nM human insulin (Novo Nordisk, Bagsværd, Denmark) for 3 hours. Cinnamaldehyde and eugenol were dissolved in dimethyl sulphoxide (DMSO), which equals a final concentration of 0.025% (v/v). DMSO-treated cells served as control.

For glycogen synthesis, cells were stimulated with C-14 glucose (0.2 μCi/μl) in parallel with human insulin for 3 hours. After precipitation with KOH and incubation with glycogen for 30 min at 95°C, the solutions were stored overnight at −20°C. Radiation was measured by liquid scintillation counting.

### Fao cell cultures

Fao cells were cultured in RPMI 1640 medium supplemented with 10% FCS, 100 U/ml penicillin, and 100 μg/ml streptomycin. For experiments, the cells were starved overnight in FCS-free medium and subsequently treated with 50 μM cinnamaldehyde, 500 μM eugenol (both Sigma Aldrich, Germany) or tinctura cinnamomi (1:2000; Maros, Germany) for 24 hours before stimulating them with 100 nM human insulin (Novo Nordisk, Bagsværd, Denmark) for 60 min.

### Animals

Male B6.V-*Lep* ^ob^/J mice on a C57BL/6J background (ob/ob) and C57BL/6J mice were purchased from the Jackson Laboratory (Bar Harbor, USA) and kept in the same specific pathogen-free facility. B6.V-*Lep* ^ob^/J mice were fed normal chow diet (Ssniff® R/M-H, Soest, Germany). Six-week-old C57BL/6J control animals were weaned on a high fat diet (HFD, Harlan Laboratories, Madison, USA) with 45% of kcals from fat (lard) for 6 weeks before starting the experiments. The fatty acid profile of this diet consisted of 36% saturated fatty acids (% of total fat). Body weight and food intake were determined weekly, and body weight gain and food intake are depicted after 6 weeks of supplementation. In parallel, drinking water was either supplemented with 4.5 ml/kg body weight (equates to 0.8 g/kg body weight) cinnamon extract (*Cinnamomum zeylanicum*, Maros, Fürth, Germany) or 70% ethanol (4.5 ml/kg body weight) as vehicle solution for 6 weeks and prepared fresh daily. The final amount of ethanol per mouse per day was around 8 μg. The animals were maintained on a 12 h light-dark cycle. All animal procedures were approved by government authorities of the University of Tuebingen (Permit Number: M2/11) for animal research according to the guidelines of laboratory animal care. Surgeries were performed under ketamine/xylazine and isoflurane anesthesia and animals got all efforts to minimize suffering.

### Electrocorticography (ECoG) measurements and locomotor activity

Radiotelemetric measurements of cortical brain activity and locomotion were performed as previously described [35]. Briefly, a telemetry electrocorticography transmitter (DSI, St Paul, MN, USA) was subcutaneously implanted under ketamine/xylazine and isoflurane anesthesia and each mouse was instrumented with an intracerebroventricular guide cannula for microinjection of substances. Two lead wires were connected to epidural placed microscrews and a sterile 27-G stainless steel cannula 6 mm in length was implanted in the left lateral ventricle of the brain. Continuous recording of telemetry signals (ECoG and locomotor activity) were conducted for the whole experimental procedure and data analysis for ECoG measurements were performed using fast Fourier transformation for delta (0.5–4 Hz), theta (4–8 Hz), alpha (8–12 Hz) and beta (12–30 Hz) frequency bands to calculate the power spectral density in μV^2^/Hz. Before starting intracerebroventricular applications, basal ECoG measurements were run during a 4-day period.

### Intracerebroventricular application of human insulin

Human insulin (Novo Nordisk, Bagsværd, Denmark) was intracerebroventricularly (i.c.v.) applicated in a concentration of 3.75 mU/5 μl (which correspond to 5.41 nmol/ml) and compared to i.c.v. given control solution (0.9% NaCl). ECoG and locomotor activity were continuously measured during 120 min post-injection.

### Glucose tolerance test and determination of homeostasis model assessment of insulin resistance (HOMA-IR)

To test glucose tolerance after the 6-week lasting treatment period, overnight fasted mice were injected with 2 g/kg body weight of α-D-glucose intraperitoneally (i.p.) and blood glucose concentrations were determined from tail bleeds after 0, 15, 30, 60, and 120 min using a Glucometer Elite (Bayer, USA). The HOMA-IR was calculated with glucose and insulin concentration obtained in overnight fasted animals using the following formula: glucose (mg/dl) x insulin (mU/ml)/405 corresponding to the computer-based HOMA2Calculator software freely available at http://www.dtu.ox.ac.uk/homacalculator/index.php; the cut-off for insulin resistance was set at HOMA-IR<2.5.

### Analysis of plasma insulin

Blood samples were kept on ice until centrifugation, and plasma was stored at −20°C. A sensitive rat insulin RIA (Millipore, USA) was used to measure plasma insulin levels.

### Magnetic resonance imaging (MRI)

MRI examinations for quantification of fat volume were performed on a 3 T whole-body imager (Magnetom Trio, Siemens Healthcare, Erlangen, Germany) after the 6-week lasting treatment period when animals reached an age of 12 weeks. Anaesthetized mice were positioned in prone position in the wrist coil of the manufacturer and MRI examinations were simultaneously performed in B6.V-*Lep* ^ob^/J (ob/ob) and C57BL/6J mice supplemented with cinnamon extract or vehicle solution. Abdominal fat images were recorded with an in-plane spatial resolution of 0.4 mm and a slice thickness of 2 mm. The fat volume was extracted as described in [18]; [36].

### Food intake, drink behavior and energy expenditure

Food and drink intake were recorded with an automated Drinking & Feeding Monitor system (TSE, Bad Homburg, Germany) consisting of bottles and baskets connected to weight sensors. Mice were habituated to the test cages for 2 days before trials, and the measurement period lasted 24 hours. Recorded data were analyzed as cumulative food and drink intake and the resulting area under the curve (AUC). Respiratory quotient (RQ) was determined by indirect calorimetry at 22°C for 24 hours in mice in an open circuitry calorimetry system (TSE). Rates of oxygen consumption (VO_2_) and carbon dioxide production (VCO_2_) were measured with a flow rate of 0.38l/min. VO_2_ and VCO_2_ were recorded for 1.5 min in 17-min intervals for each animal. Recorded data were analyzed as mean RQ for each hour and displayed as a time course of 24 hours and the corresponding AUC.

### Analysis of triglycerides, cholesterol and lipoproteins

All clinical chemical parameters (total-, HDL- and LDL-cholesterol and triglycerides) were measured on the automated clinical chemistry analyzer ADVIA 1800 (Siemens Healthcare Diagnostics, Eschborn, Germany). To determine the hepatic triglyceride content, livers were homogenized in PBS containing 1% Triton X-100 using a TissueLyser (QIAGEN Sciences, Germantown, MD). Triglycerides of liver homogenates were quantified using the ADVIA 1800 clinical chemistry analyzer (Siemens Healthcare Diagnostics, Eschborn, Germany) and normalized to wet tissue weight.

### Western Blot analysis

A bolus of human insulin (Novo Nordisk, Denmark; 1U/mouse for 10 min) or a comparable amount of saline was injected into the inferior vena cava of overnight fasted mice under ketamine/xylazine anaesthesia. After decapitation, total brain and liver tissues were quickly removed and homogenized in lysis-buffer as previously described [37]. Equivalent protein amounts were immunoprecipitated with antibody directed against insulin receptor (IR) to detect tyrosine phosphorylation [38]. Visualization of immunocomplexes was accomplished after gel electrophoresis and Western blot experiments using antibodies directed against phosphotyrosine PY-20 (Santa Cruz Biotechnology, Heidelberg, Germany) and P-AKT (Ser473) (Upstate, Charlottesville, USA). In liver lysates, phospho-STAT3 antibody (Tyr705; Cell Signaling Technology; Millipore, Billerica, MA, USA) and the nonradioactive enhanced chemiluminescence system ECL was used. For Western blot essays in Fao cells antibodies raised against phospho-AKT, phospho-glycogen synthase kinase-3 (GSK3) and phospho-signal transducer and activator of transcription 3 (STAT3) were applied (all Upstate, Charlottesville, VA, USA). Western blot experiments in astrocytes were performed in lysates using antibodies raised against phospho-GSK3α/β (Upstate, Charlottesville, VA, USA), P-AKT (Ser473), and protein tyrosine phosphatase 1B (PTP-1B) (Santa Cruz Biotechnology, Heidelberg, Germany). Tubulin and GAPDH (Cell Signaling Technology; Millipore, Billerica, MA, USA) served as loading control. Optical densitometry was performed using ImageJ software (freely available at http://rsb.info.nih.gov/ij/index.html) to compare bands in autoradiographs.

### Hepatic glycogen content

Hepatic glycogen content was determined by a modification of the procedure described by Chan and Exton [39]. Weighted liver tissue was solubilized for 10 min with 250 μl 30% KOH at 90°C. After adding 0.2 volumes 1 M Na_2_SO_4_ and 3 volumes 100% ice-cold ethanol glycogen was precipitated by centrifugation (30 min, 10.000×*g*, 4°C). The precipitate was washed twice with 70% ethanol, dried and hydrolyzed with 300 μl 1 M HCl at 90°C for 1 h. After neutralization with 1 M NaOH and 10 mM HEPES (pH 7.5), glucose was determined enzymatically using hexokinase in an Advia 1650 system.

### Statistical analysis

Data are expressed as mean±SEM of the indicated number of experiments and analyzed using Origin 8.1 (Northampton, MA, USA) software. Significance analysis was performed using Student's t test and analysis of variance (ANOVA) with Dunnett's or Bonferroni's post-hoc test for multiple comparison analysis. Significance was set at *P*<0.05.

## Results

### Altered responses on glycogen formation and insulin signaling by cinnamaldehyde and eugenol in cultured primary astrocytes

**Figure 1:**
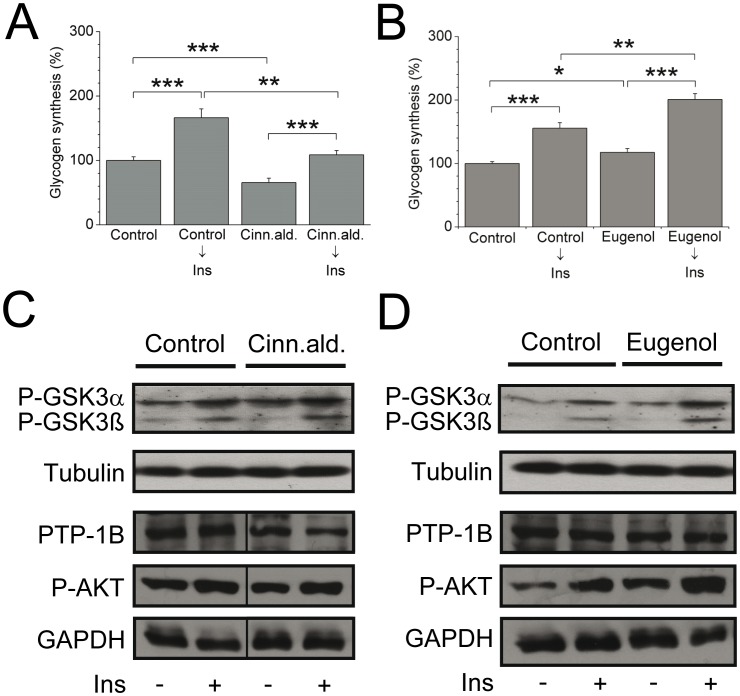
Effect of cinnamaldehyde and eugenol in cultured primary astrocytes. A,B: Glycogen synthesis in mouse astrocytes treated with insulin, 50 μM cinnamaldehyde (Cinn.ald.) (A), 500 μM eugenol (B) or the substances followed by 100 nM insulin (*n* = 12/group). Significance as indicated: *P<0.05; **P<0.005, ***P<0.001. Data are mean ± SEM. C,D: Insulin-stimulated phosphorylation of GSK3α/β and AKT, and protein expression of PTP-1B after treatment with 50 μM cinnamaldehyde (C) and 500 μM eugenol (D). A representative Western Blot is shown out of 5 (*n* = 4–7 replications per condition) independent experiments. Tubulin or GAPDH was used as loading control. Ins, insulin.

To test a potential brain effect of cinnamon, we first performed an experiment to study the *in vitro* effect of two major components of cinnamon, cinnamaldehyde and eugenol on isolated primary murine astrocytes. This cell type is the most abundant in the brain and responsible for providing nutrients for neurons. We recently demonstrated that astrocytes are insulin responsive and form glycogen upon insulin stimulation [33]. Cinnamaldehyde significantly reduced glycogen formation compared to control in the non-stimulated condition, and significantly diminished insulin-stimulated glycogen formation (Fig. 1A). By contrast, eugenol significantly promoted insulin-mediated glycogen formation in primary astrocytes (Fig. 1B), while co-treatment with cinnamaldehyde attenuated this effect (data not shown). On the insulin signaling cascade, cinnamaldehyde did neither affect phosphorylation of GSK3 nor P-AKT, and no changes in PTP-1B expression could be detected (Fig. 1C). By contrast, eugenol enhanced phosphorylation of GSK3, but did not affect PTP-1B (Fig. 1D). Notably, eugenol ameliorated phosphorylation of AKT by 76% (non-stimulated: 0.97±0.19 vs. 0.55±0.15 arb. unit) and 41% (insulin-stimulated: 1.38±0.24 vs. 0.98±0.15 arb. unit) compared to control condition (Fig. 1D). These results demonstrate that two major compounds of cinnamon exert different effects in astrocytes suggesting that eugenol is an active component of cinnamon extract to enhance insulin signaling in the brain. This prompted us to investigate the effect of cinnamon extract in animal models.

### Cinnamon extract supplementation ameliorates insulin sensitivity in the brain and results in increased cortical and locomotor activity in B6.V-*Lep* ^ob^/J (ob/ob) mice

**Figure 2:**
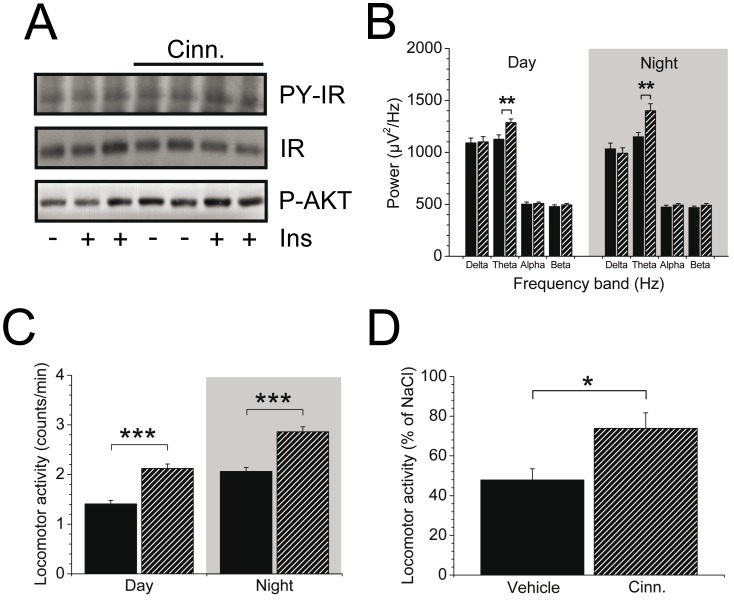
Impact of cinnamon extract supplementation on insulin sensitivity in the brain and the influence on cortical and locomotor activity in B6.V-*Lep* ^ob^/J (ob/ob) mice. A: Representative Western Blot out of 3 independent experiments of tyrosine phosphorylation (PY) of IR and AKT (S473) and protein expression of IR after intravenous insulin injection in overnight-fasted cinnamon extract-supplemented (Cinn.) ob/ob mice (*n* = 2–5 replications per condition). B: Analysis of basal power spectral density (indicated for delta (0.5–4 Hz), theta (4–8 Hz), alpha (8–12 Hz), and beta (12–30 Hz) frequencies) using fast Fourier transformation and estimated by ECoG in ob/ob mice supplemented with cinnamon extract (cross-hatched) or vehicle solution (filled). Basal ECoG measurements were conducted during a 4-day period (*n* = 7–8/group; **P<0.005 between cinnamon or vehicle treatment. C: Averaged locomotor activity after cinnamon (cross-hatched) or vehicle (filled) supplementation during the day (7 AM to 7 PM) and night (7 PM to 7 AM) cycle from a 4-d period (***P<0.001). D: Effect of acute intracerebroventricular (i.c.v.) insulin application (indicated as % of i.c.v. injected NaCl solution) on activity in cinnamon extract (cross-hatched) treated ob/ob mice compared to vehicle treatment (filled). *P<0.05 to vehicle supplementation. Data are presented as mean ± SEM. Ins, insulin.

We further evaluated the brain response to insulin in cinnamon treated ob/ob mice. Western Blot analysis revealed that phosphorylation of insulin receptor and AKT was slightly improved due to chronic cinnamon administration. Moreover, in the cinnamon group, tyrosine phosphorylation of AKT was higher in the basal state compared with vehicle treated mice, and insulin-stimulated P-AKT implicated an enhancement of 32% compared to levels of vehicle treated mice (138.7±12.6 vs. 105.4±13.7 arb. unit) (Fig. 2A). As previous studies revealed that insulin sensitivity in the brain is accompanied by an increase in cortical activity, we performed analysis of brain activity by using telemetric implants. Interestingly, cortical activity in ob/ob mice was solely increased in the theta frequency band (Fig. 2B). Because of this, we considered alterations in locomotor activity [17] and assessed random and insulin-mediated locomotion. As expected, ob/ob mice were less active during the day and night phase but cinnamon treatment was highly sufficient to increase random locomotor activity in ob/ob mice during day and night when theta activity is significantly increased (Fig. 2C).

As an alternative measure of insulin sensitivity in the brain, insulin-mediated locomotion was evaluated. Mice received an intracerebroventricular injection of either insulin or physiological NaCl solution and locomotor activity was detected by telemetric implants. Interestingly, locomotion was significantly reduced in ob/ob mice but greatly improved due to insulin stimulation in mice that received cinnamon extract (Fig. 2D). These data are consistent with our hypothesis that cinnamon extract directly promotes insulin action in the brain and exerts beneficial effects on cortical activity and locomotion.

In terms of sleep architecture, cinnamon treated ob/ob mice were slightly more awake during the night and day time (night: 115±13 min vs. 100±17 min; day: 87±11 min vs. 73±11 min, *P*<0.05).

### Cinnamon extract supplementation results in improved insulin sensitivity in the brain without any beneficial metabolic consequences in HFD-fed C57BL/6 mice

In HFD-fed mice, the brain response to insulin was also ameliorated by cinnamon supplementation (Suppl. Fig. S1), and Western Blot analysis revealed that tyrosine phosphorylation of insulin receptor and AKT was improved and insulin-stimulated PTP-1B expression was diminished due to chronic cinnamon administration (Suppl. Fig. S1).

Because the brain is the key organ to sense metabolic alterations and in turn controls food intake and glucose metabolism, we next measured glucose homeostasis in mice supplemented with cinnamon extract for 6 weeks. In HFD-fed mice, cinnamon extract was not sufficient to significantly affect body weight (Suppl. Fig. S2A). Because cinnamon extract was shown to increase uptake of glucose into 3T3-L1 adipocytes and increase the expression of peroxisome proliferator-activated receptor gamma (PPAR-γ) *in vitro*, we went on to assess fat mass in the respective animals. Fat accumulation and in particular visceral adipose tissue was not altered due to cinnamon extract in HFD-fed mice as determined by magnet resonance imaging techniques (Suppl. Fig. S2B). In addition, cinnamon did not affect fasted plasma blood glucose levels (Suppl. Fig. S2C) and HOMA-IR (Suppl. Fig. S2D) in HFD-fed mice. To further test the specific effect of cinnamon on glucose tolerance and insulin secretion, we performed intraperitoneal glucose tolerance tests. Cinnamon was not effective to lower blood glucose in normoglycemic HFD-fed mice (Suppl. Fig. S2E), and insulin secretion after the glucose load was similar to control supplementation (Suppl. Fig. S2F).

### Metabolic consequences of cinnamon extract supplementation in B6.V-*Lep* ^ob^/J (ob/ob) mice

**Figure 3:**
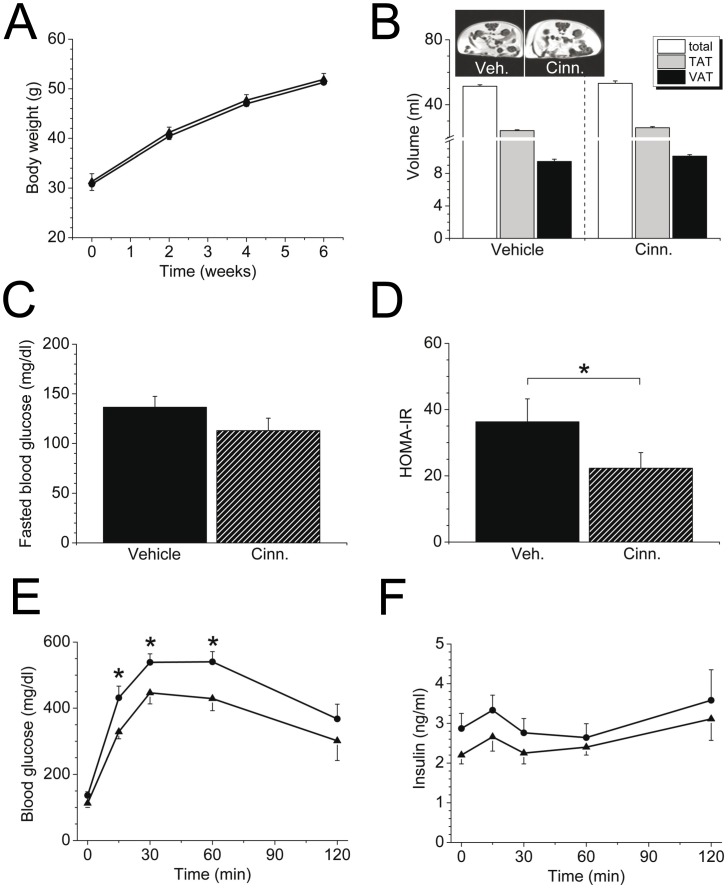
Metabolic consequences of cinnamon extract supplementation in chow-fed B6.V-*Lep* ^ob^/J (ob/ob) mice. Ob/ob mice were supplemented with cinnamon extract or vehicle solution for 6 weeks (*n* = 10/group). A: Body weight development during the supplementation period with cinnamon extract (triangles) or vehicle solution (dots). B: Magnetic resonance images of total (TAT, grey) and visceral fat (VAT, black) deposits in ob/ob mice supplemented with cinnamon extract (Cinn.) or vehicle solution (Veh.) for 6 weeks. Calculated volumes of TAT and VAT integrated over 24 slices are quantified of *n* = 6 mice per supplemented group. Insert: Bright (hyperintense) areas represent fat tissue. C: Fasted blood glucose concentrations of cinnamon extract- (cross-hatched) or vehicle- (filled) treated ob/ob mice after the 6 week treatment period. D: Calculated HOMA-IR in the feed-deprived state; *P<0.05. E: Blood glucose in i.p. GTT in cinnamon extract-treated (triangles) and vehicle-treated (filled dots) ob/ob mice (*P<0.05). F: Plasma insulin concentration during the GTT after cinnamon extract (triangles) or vehicle (dots) treatment. Data are presented as mean ± SEM.

As expected, body weight gain was markedly enhanced in ob/ob mice compared to HFD-fed mice (*P*<0.001), whereas cinnamon was not able to counteract the body weight increase of around 20 g during the 6-week-long supplementation period (Fig. 3A). Accordingly, no change in body fat mass in ob/ob mice was detected by cinnamon extract (Fig. 3B). Additionally, gene expression of PPAR-γ, fatty acid synthase, sterol regulatory element binding protein (SREBP), monocyte chemoattractant protein-1 (MCP-1), interleukin 6 (IL-6), interleukin 1 beta (IL-1β) or sirtuin 1 (SIRT1) in fat tissues was not altered due to cinnamon treatment (data not shown). In agreement with previous reports, fasting blood glucose concentrations were lower in diabetic mice (Fig. 3C, *P* = 0.059) and HOMA-IR was significantly decreased in ob/ob mice that received cinnamon extract (Fig. 3D), implying that cinnamon promotes glucose homeostasis and insulin sensitivity *in vivo*. Notably, cinnamon extract greatly improved glucose tolerance in heavily obese and diabetic ob/ob mice (Fig. 3E): however, insulin secretion in an intraperitoneal glucose tolerance test was comparable between groups (Fig. 3F) despite lower fasting insulin levels as a measure of improved insulin sensitivity in cinnamon treated ob/ob mice (*P* = 0.14).

### Food intake, energy expenditure and respiratory quotient of cinnamon extract-supplemented HFD-fed C57BL/6 and chow-fed B6.V-*Lep* ^ob^/J (ob/ob) mice

**Figure 4:**
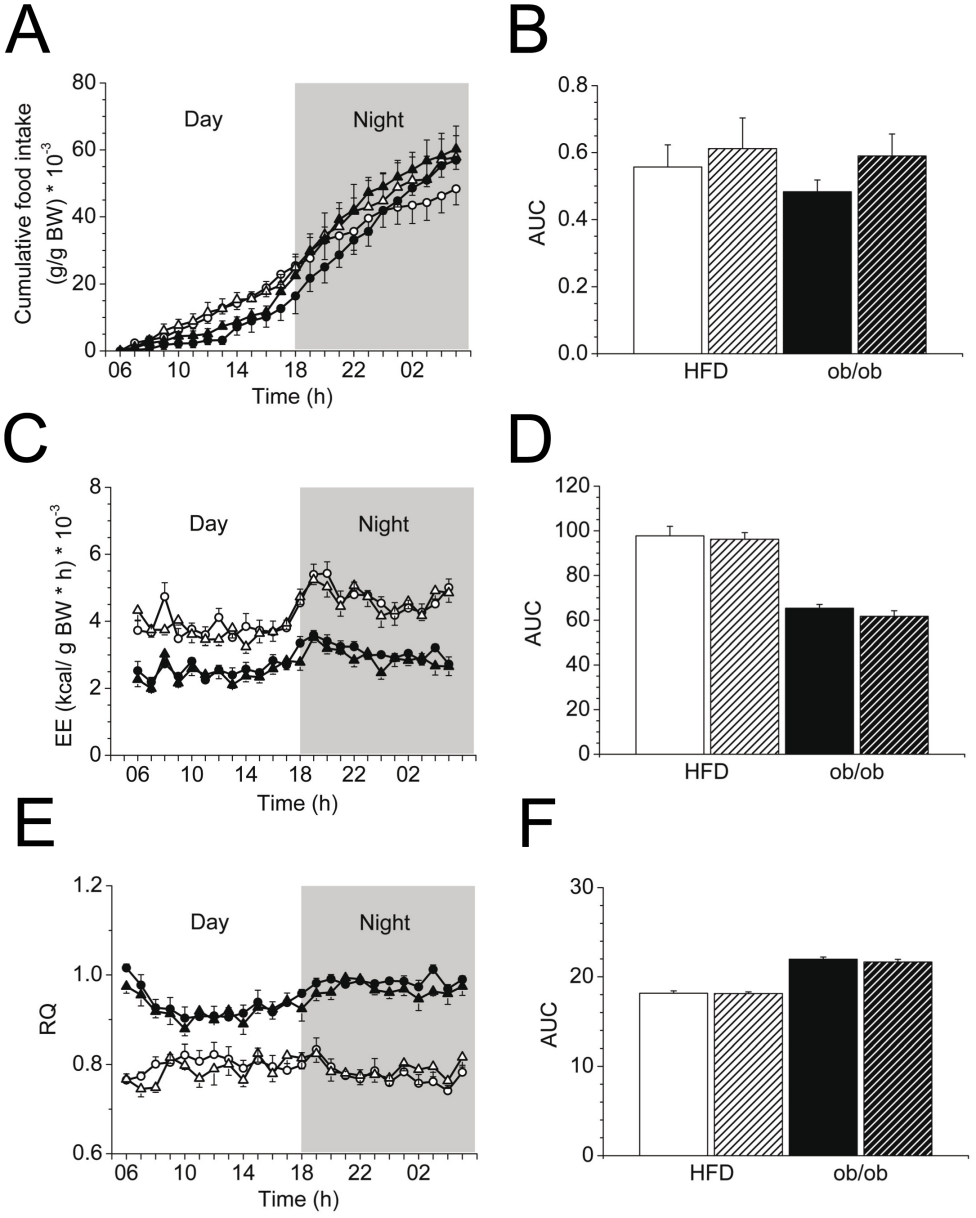
Food intake, energy expenditure and respiratory quotient of cinnamon extract-supplemented HFD-fed C57BL/6 and chow-fed B6.V-*Lep* ^ob^/J (ob/ob) mice. A,B: Food intake related to body weight after 6-week-long supplementation with cinnamon extract (triangles, cross-hatched) or vehicle (dots, filled) solution in HFD-fed (white) and ob/ob (black) mice shown as 24-hour-long measurement period (A) or area under the curve (AUC) for the 24 hours average (B), *n* = 6/group. C,D: Energy expenditure (kcal/g body weight * h) over 24 hours (C) and as average depicted as AUC (D). E,F: Respiratory quotient of the abovementioned animals over a 24 hours period (E) and corresponding AUC (F); *n* = 6/group. All data are represented as mean ± SEM. EE: energy expenditure; RQ: respiratory quotient.

To gain insight into the role of cinnamon supplementation in body energy homeostasis, we further analyzed food intake after the 6-week-long supplementation period. No effect in food intake was detected, either in HFD-fed control or ob/ob mice (Fig. 4 A,B). Additionally, we monitored energy expenditure and respiratory quotient for a 24 h period in the respective mice. As expected, a reduced energy expenditure was determined in ob/ob mice compared to HFD-fed controls, but cinnamon supplementation had no beneficial effect (Fig. 4 C,D). Further, the respiratory quotient of cinnamon-treated groups did not differ to the vehicle-treated groups (Fig. 4 E,F). These data suggest that cinnamon supplementation did not trigger a negative energy balance by suppressing energy intake or stimulating energy expenditure.

### Cinnamon extract lowers hepatic fat in B6.V-*Lep* ^ob^/J (ob/ob) mice

**Figure 5:**
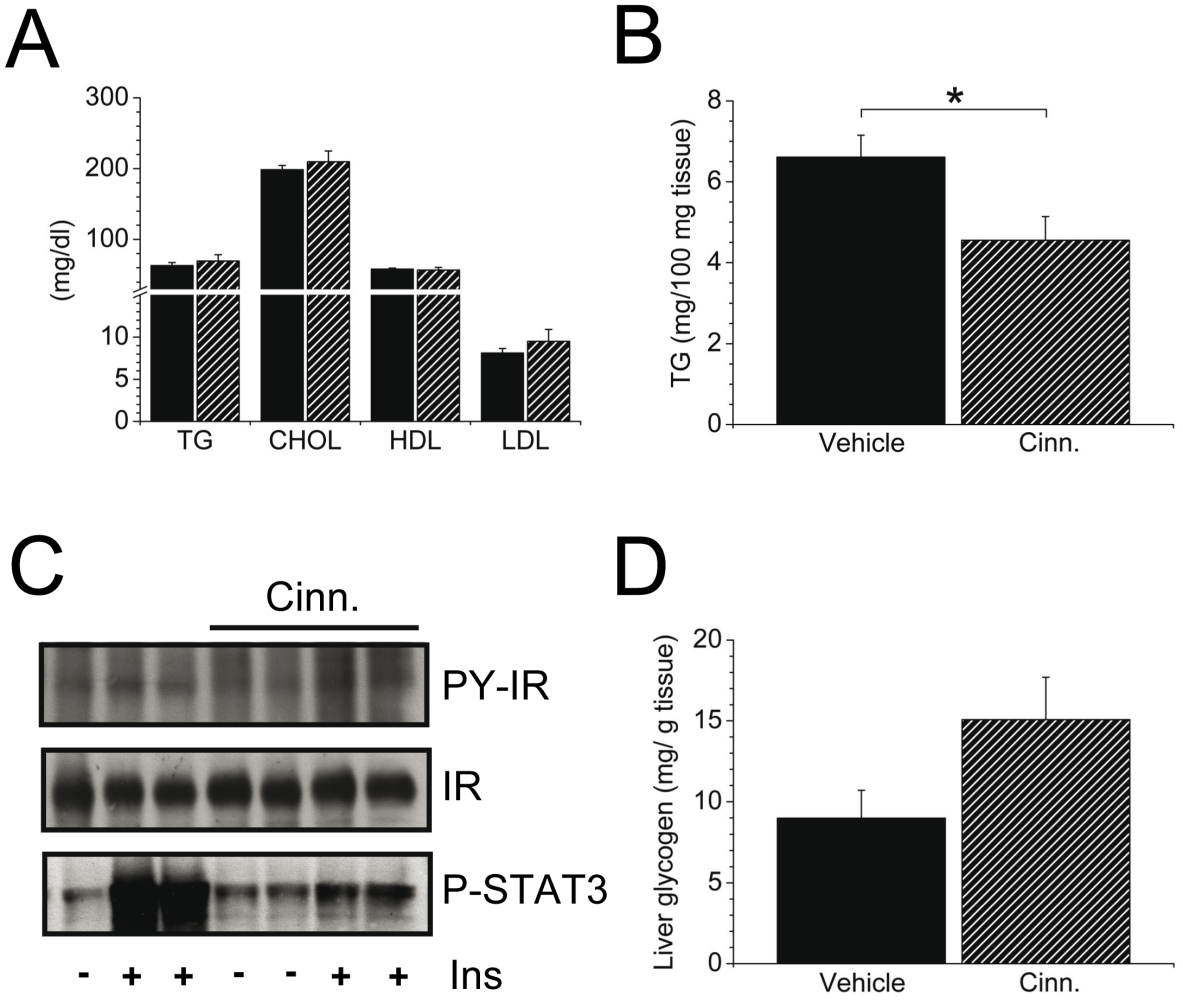
Effect of cinnamon extract supplementation on liver and serum parameters in B6.V-*Lep* ^ob^/J (ob/ob) mice. A: Triglycerides (TG), cholesterol (CHOL), HDL and LDL concentration in plasma of ob/ob mice (*n* = 6/group) supplemented with cinnamon extract (cross-hatched) or vehicle solution (filled). B: Triglyceride content (mg/100 mg tissue) in liver tissue of ob/ob mice supplemented with cinnamon extract or vehicle solution in the fasted state (*n* = 6/group; *P<0.05). C: Western Blot analysis in liver tissues after intravenous insulin injection in overnight fasted animals; *n* = 2 replications per condition). A representative Western Blot is shown out of 2 independent experiments. Cinn., cinnamon-supplemented animals; Ins, insulin. D: Liver glycogen concentration (mg/g tissue) after cinnamon (Cinn.) supplementation in ob/ob mice (*n* = 7–10/group). Data are mean ± SEM.

When we further assessed plasma parameters in ob/ob mice, no changes in cholesterol and triglyceride plasma concentrations were determined by cinnamon treatment (Fig. 5A). Moreover, cytokine concentrations in the plasma like IL-1β, IL-6, IL-10, IFN-γ, TNF-α, RANTES and MCP-1 were indistinguishable between the cinnamon-supplemented and control groups (data not shown).

To assess whether fat accumulation in the liver was affected by cinnamon extract, triglyceride content in liver tissues was determined. Ob/ob mice are characterized by hepatic steatosis and cinnamon treatment was sufficient to suppress fat accumulation in the liver substantially (Fig. 5B). As liver steatosis is commonly associated with insulin resistance [40], we assessed insulin receptor phosphorylation in liver tissues *in vivo*. In profoundly insulin resistant ob/ob animals, cinnamon promoted tyrosine phosphorylation of the insulin receptor in liver tissues (insulin-stimulated PY-IR: 61.3±3.3 vs. 35.4±5.3 arb. unit in cinnamon group and 36.7±3.5 vs. 40.1 arb. unit in vehicle group (compared to non-stimulated conditions)). As STAT3 is important for glucose homeostasis and operates as hepatic effector of insulin's action in the brain we further assessed its phosphorylation. Remarkably, an increase of STAT3 phosphorylation in the basal state was detected by cinnamon supplementation (Fig. 5C). However, these alterations did not translate to changes in liver enzymes, and gene expression analysis revealed no change in IL-6, IL-1β or TNF-α in liver tissues harvested from cinnamon treated ob/ob mice compared to control supplementation (data not shown).

Due to decreased hepatic triglyceride levels by cinnamon treatment one might assume that energy reserves are shunted into the production of glycogen as quickly oxidizable energy source, and indeed, ob/ob mice exhibited an increase of 68% (*P* = 0.09) in hepatic glycogen concentration due to cinnamon supplementation compared to vehicle-treated ob/ob mice (Fig. 5D).

### Cinnamaldehyde, eugenol, and cinnamon extract do not interfere with insulin signaling in Fao cells

**Figure 6:**
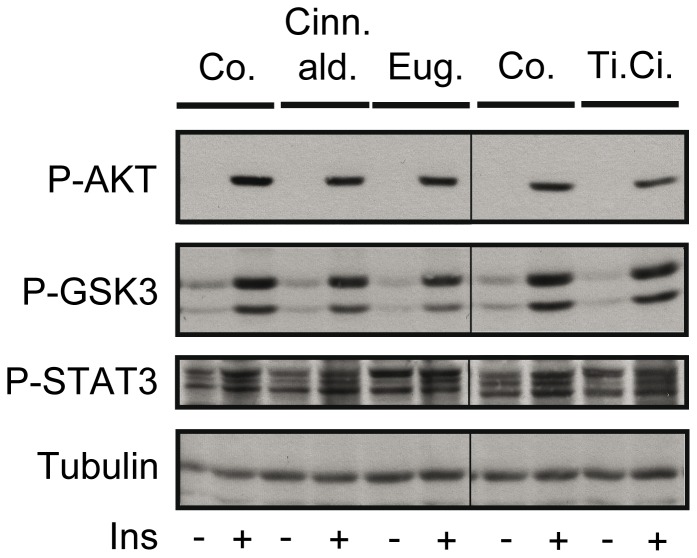
Impact of cinnamaldehyde, eugenol, and cinnamon extract on insulin signaling in Fao cells. Fao cells were pretreated for 24 μM cinnamaldehyde (Cinn.ald.), 500 μM eugenol (Eug.) and cinnamon extract (tinctura cinnamomi ((Ti.Ci.)) (1:2000) before stimulating them with 100 nM insulin (Ins) for 60 min. After lysis, cells were separated by SDS-PAGE and analyzed for the phosphorylation of AKT, GSK3, and STAT3. A representative Western Blot is shown out of 2 independent experiments. Tubulin was used as loading control.

To reveal if hepatic changes by cinnamon supplementation might be ascribed to the liver itself or to brain-insulin action on liver, we analyzed the effect of cinnamon supplementation on insulin signaling in the differentiated rat hepatoma line Fao. Fao cells were treated both with cinnamaldehyde and eugenol, two major compounds of cinnamon extract, and the extract itself. Compared to control solutions, no changes in phosphorylation of AKT, GSK3 and STAT3 were detected (Fig. 6).

## Discussion

Diabetes originates from impaired insulin action in target tissues followed by inadequate insulin secretion. Genetic predisposition, environmental factors such as physical inactivity and exposure to high caloric food drive the progression of the disease. Obesity is among the most abundant factors in impairing glucose homeostasis and there is a need for therapies that fight insulin resistance and fatty liver in obese patients at risk for type 2 diabetes.

While the adherence to present therapeutic regimes is rather poor most likely due to the complexity of the regime, and the risk for hypoglycemia and other side effects, there is a need for complementary and alternative therapies for the treatment of diabetes. Among others, cinnamon came up as a nutraceutical to improve glycemic control in humans without documented potential toxic effects and a high therapeutic window. In terms of glucose metabolism, at least the short term effects of cinnamon looked promising [41]–[42]. Even a reduction in cardiovascular disease and the risk for colonic cancer as well as boosting cognitive function were demonstrated most likely due to an improvement in insulin sensitivity [1]. It was reported that the intake of cinnamon powder for 4 months significantly reduced fasting plasma glucose concentrations, triglyceride and LDL-cholesterol levels [43].

This study provides *in vivo* evidence that cinnamon extract beneficially affects brain activity and glucose homeostasis in mouse models of obesity and diabetes. Recently, we and others demonstrated evidence for a crucial role of astrocytes in the pathogenesis of insulin resistance and altered brain activity. Astrocytes are indispensable to form and stabilize synapses, and they regulate the concentration of various molecules to support energy metabolism for neurons [44]. Primary murine astrocyte cultures are a well-established cell system for detecting changes in glycogen synthesis [31]–[33], and by using these we demonstrated altered responses on glycogen formation and insulin signaling by specific compounds of cinnamon extract. Importantly, our data demonstrated a favorable effect of eugenol on basal and insulin-mediated glycogen synthesis in primary mouse astrocytes, whereas cinnamaldehyde acted differently and had even an inhibitory effect on basal glycogen synthesis. This inhibition was also true when cinnamaldehyde was additionally applied to eugenol pointing up a putative negative role of cinnamaldehyde on providing neighboring neurons or axons with fuel. In the context of negative regulators of central leptin and insulin signaling, PTP-1B plays a key role in the regulation of insulin sensitivity by dephosphorylation of the insulin receptor and its downstream signaling components [45]–[46]. In this study, however, neither cinnamaldehyde nor eugenol treatment affected PTP-1B expression in murine astrocytes. These data suggest that eugenol exerts insulin sensitizing effects via phosphorylation of GSK3 and AKT but not via PTP-1B. Eugenol was reported to protect neuronal cells from excitotoxic and oxidative injury in primary cortical cultures and seems to be one of the effective components in cinnamon extract [47]–[48]. Because the impact and interplay of specific cinnamon compounds on whole body metabolism is unknown, we carried on to investigate the effect of cinnamon extract on the whole in a diabetic and obese animal model.

The brain plays an important role in sensing metabolic alterations and in controlling food intake and glucose homeostasis. These regulatory mechanisms are impaired by obesity. We have previously shown that obese animals and humans display insulin resistance in the brain which translates to alterations in hepatic gluconeogenesis, insulin sensitivity in the periphery, locomotor activity and finally glucose disposal [49]. In this study approach, cinnamon was able to improve insulin sensitivity in insulin resistant brains of ob/ob mice. This was accompanied by an increase in basal cortical and locomotor activity. In previous studies, we established the insulin-mediated increase in theta activity and locomotion as a measure of insulin sensitivity in the brain [17], and this correlation could be confirmed in the present study. Thereby it became evident that insulin responsiveness improved due to cinnamon treatment with its beneficial effect on brain activity and locomotion. In particular, cinnamon extract let obese mice move more and made them more awake.

Consistent with the ameliorated HOMA-lR, we also found that ob/ob mice supplemented with cinnamon extract were characterized by decreased blood glucose levels after an intraperitoneally applied glucose bolus. These favorable changes in glucose homeostasis together with elevated insulin sensitivity in the brain may contribute to amended brain activity by cinnamon extract supplementation. Notably, cinnamon was not able to lower blood glucose levels in normoglycemic animals to hypoglycemia and this is in agreement with studies in humans where cinnamon was effective in patients with type 2 diabetes but not reported in healthy controls. In diabetic animals, cinnamon attenuated weight loss that is associated with hyperglycemia, but there was no net weight effect obtained [22]. This is in line with our data where cinnamon was not able to affect body weight gain, as well as food intake and energy expenditure. Although, one might speculate on a weight sparing effect in physically active ob/ob mice and an attenuate weight loss due to normoglycemic glucose levels in the cinnamon treated group.

In our models, fasting glucose levels decreased in heavily obese and insulin resistant mice by cinnamon extract supplementation for 6 weeks. To further dissect the favorable effect of cinnamon on fasting blood glucose levels [50], we investigated hepatic fat, glycogen content and insulin sensitivity in the liver. In our diabetic and insulin resistant model, a decrease in liver triglyceride content and an increase in glycogen concentration in the cinnamon-treated ob/ob group were observed and this went along with improved insulin sensitivity of the liver. These results are in agreement with a previous study reporting that cinnamon intake augmented hepatic glycogen levels in high fat/high fructose diet-fed rats [51]. Notably, gene expression profiles in liver tissues were not able to document a direct effect of cinnamon on liver tissues, suggesting that the observed hepatic changes *in vivo* were due to a modulation of central regulatory circuits. Besides the favorable effects of cinnamon extract on fasting blood glucose, insulin sensitivity and liver fat, we therefore reasoned that the brain plays a crucial role in the regulation of peripheral glucose homeostasis by the brain-liver axis in our model, and STAT3 is important for glucose homeostasis and represents an effector of insulin action in the brain on liver tissues [52].

However, one might take into consideration that other parts as the complex catechins or the terpene alcohol linalool are involved in the present findings, as the latter substance was shown to restore glucose-metabolizing enzymes in streptozotocin (STZ)-induced diabetic rats [53], and is involved in the attenuation of hyperglycemia in diabetic rats [54].

Together, our data suggest that treatment with cinnamon extract may exert important and yet unexplored effects to improve glucose homeostasis and liver fat content in obese and diabetic animals, and eugenol may represent the relevant compound to mediate this effect.

## Supporting Information

Figure S1 **Impact of cinnamon extract supplementation on insulin sensitivity in the brain in HFD-fed C57BL/6 mice.** Representative Western Blot out of 3 independent experiments of tyrosine phosphorylation (PY) of IR and AKT (S473) and protein expression of IR and PTP-1B after intravenous insulin injection in overnight-fasted cinnamon extract-supplemented (Cinn.) HFD-fed mice; *n* = 2–5 replications per condition. Ins, insulin.
(TIF)

Figure S2 **Metabolic consequences of cinnamon extract supplementation in HFD-fed C57BL/6 mice.** HFD-fed C57BL/6 mice were supplemented with cinnamon extract or vehicle solution for 6 weeks (*n* = 10/group). A: Body weight development during the supplementation period with cinnamon extract (triangles) or vehicle solution (dots). B: Magnetic resonance images of total (TAT, grey) and visceral fat (VAT, black) deposits in HFD-fed control mice supplemented with cinnamon extract (Cinn.) or vehicle solution (Veh.) for 6 weeks. Calculated volumes of TAT and VAT integrated over 24 slices are quantified of *n* = 6 mice per supplemented group. Insert: Bright (hyperintense) areas represent fat tissue. C: Fasted blood glucose concentrations of cinnamon extract- (cross-hatched) or vehicle- (filled) treated HFD-fed mice after the 6 week treatment period. D: Calculated HOMA-IR in the feed-deprived state. E: Effect of cinnamon extract supplementation (triangles) on plasma blood glucose levels during an i.p. glucose tolerance test compared to vehicle supplementation (dots). F: Plasma insulin concentration during the GTT in HFD-fed mice after cinnamon extract (triangles) or vehicle (dots) treatment. Data are presented as mean ± SEM.
(TIF)
