# Supplementary figures and images for: Cinnamon Extract Improves Insulin Sensitivity in the Brain and Lowers Liver Fat in Mouse Models of Obesity
Source: PLoS One. 2014 Mar 18;9(3):e92358. doi: 10.1371/journal.pone.0092358 (PMC3958529; doi:10.1371/journal.pone.0092358)

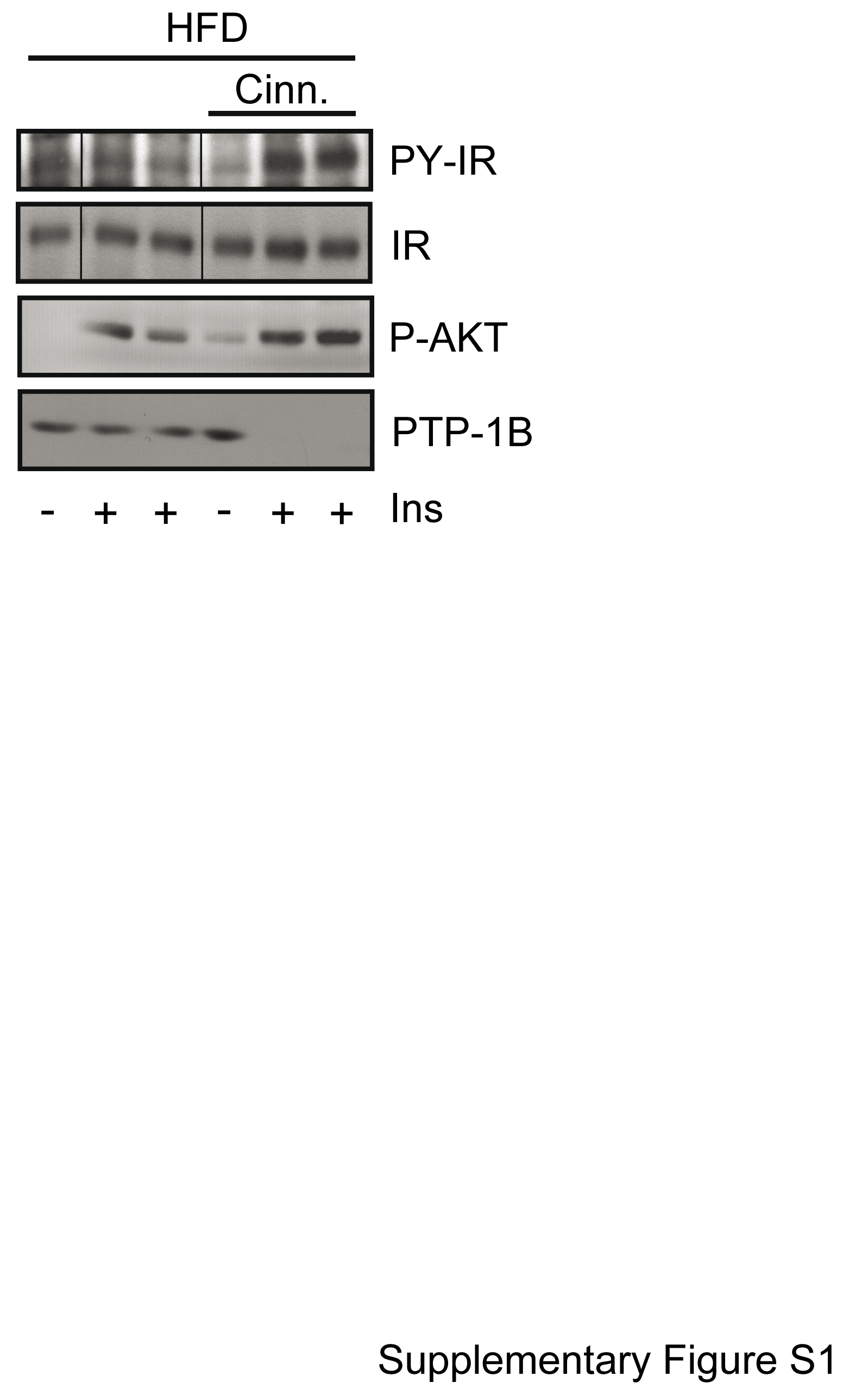

Supplement: Figure S1 — Impact of cinnamon extract supplementation on insulin sensitivity in the brain in HFD-fed C57BL/6 mice. Representative Western Blot out of 3 independent experiments of tyrosine phosphorylation (PY) of IR and AKT (S473) and protein expression of IR and PTP-1B after intravenous insulin injection in overnight-fasted cinnamon extract-supplemented (Cinn.) HFD-fed mice; n = 2–5 replications per condition. Ins, insulin. (TIF) [file pone.0092358.s001.tif]

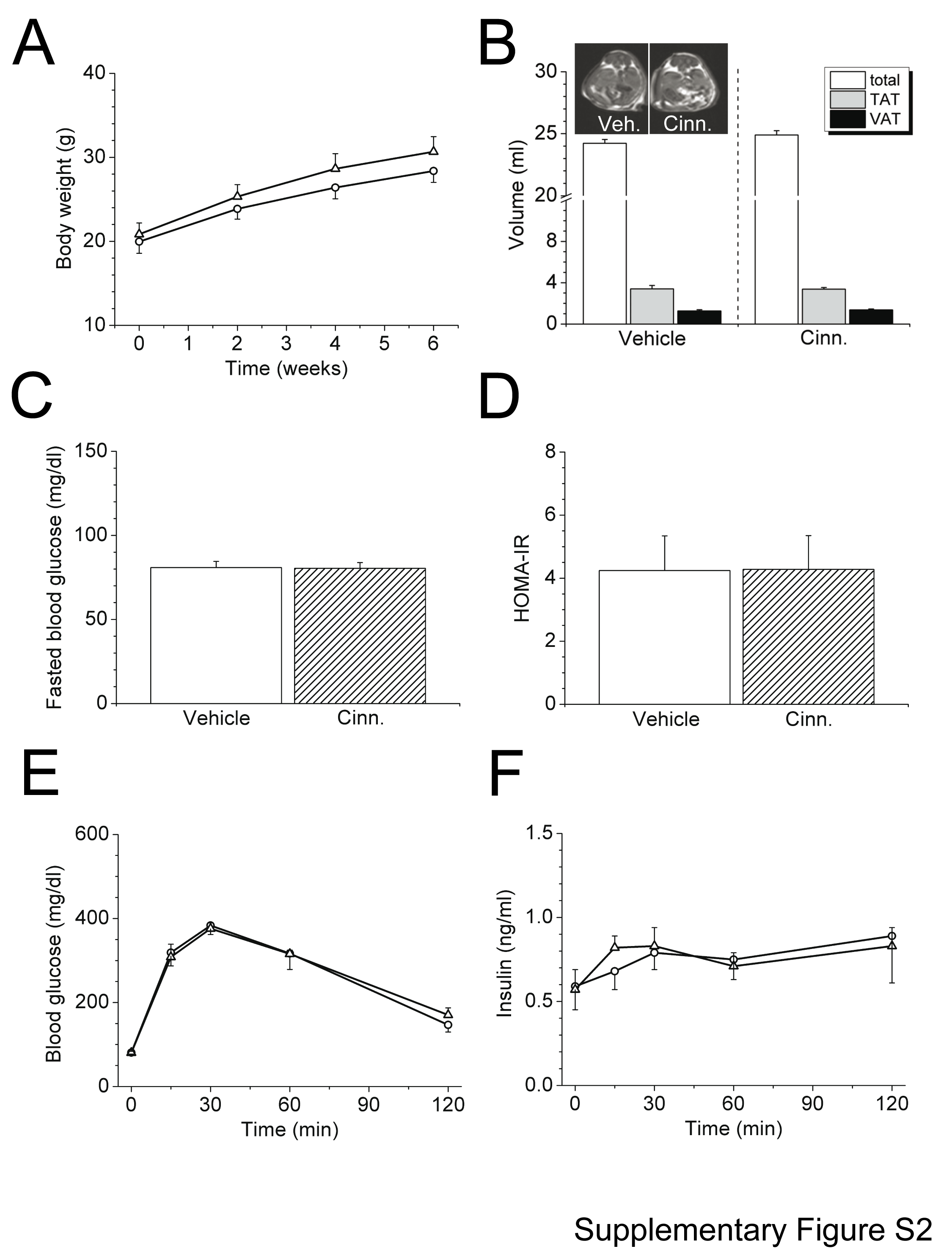

Supplement: Figure S2 — Metabolic consequences of cinnamon extract supplementation in HFD-fed C57BL/6 mice. HFD-fed C57BL/6 mice were supplemented with cinnamon extract or vehicle solution for 6 weeks (n = 10/group). A: Body weight development during the supplementation period with cinnamon extract (triangles) or vehicle solution (dots). B: Magnetic resonance images of total (TAT, grey) and visceral fat (VAT, black) deposits in HFD-fed control mice supplemented with cinnamon extract (Cinn.) or vehicle solution (Veh.) for 6 weeks. Calculated volumes of TAT and VAT integrated over 24 slices are quantified of n = 6 mice per supplemented group. Insert: Bright (hyperintense) areas represent fat tissue. C: Fasted blood glucose concentrations of cinnamon extract- (cross-hatched) or vehicle- (filled) treated HFD-fed mice after the 6 week treatment period. D: Calculated HOMA-IR in the feed-deprived state. E: Effect of cinnamon extract supplementation (triangles) on plasma blood glucose levels during an i.p. glucose tolerance test compared to vehicle supplementation (dots). F: Plasma insulin concentration during the GTT in HFD-fed mice after cinnamon extract (triangles) or vehicle (dots) treatment. Data are presented as mean ± SEM. (TIF) [file pone.0092358.s002.tif]
